# GLSNN: A Multi-Layer Spiking Neural Network Based on Global Feedback Alignment and Local STDP Plasticity

**DOI:** 10.3389/fncom.2020.576841

**Published:** 2020-11-12

**Authors:** Dongcheng Zhao, Yi Zeng, Tielin Zhang, Mengting Shi, Feifei Zhao

**Affiliations:** ^1^Research Center for Brain-Inspired Intelligence, Institute of Automation, Chinese Academy of Sciences, Beijing, China; ^2^School of Artificial Intelligence, University of Chinese Academy of Sciences, Beijing, China; ^3^Center for Excellence in Brain Science and Intelligence Technology, Chinese Academy of Sciences, Beijing, China; ^4^National Laboratory of Pattern Recognition, Institute of Automation, Chinese Academy of Sciences, Beijing, China

**Keywords:** SNN, plasticity, brain, local STDP, global feedback alignment

## Abstract

Spiking Neural Networks (SNNs) are considered as the third generation of artificial neural networks, which are more closely with information processing in biological brains. However, it is still a challenge for how to train the non-differential SNN efficiently and robustly with the form of spikes. Here we give an alternative method to train SNNs by biologically-plausible structural and functional inspirations from the brain. Firstly, inspired by the significant top-down structural connections, a global random feedback alignment is designed to help the SNN propagate the error target from the output layer directly to the previous few layers. Then inspired by the local plasticity of the biological system in which the synapses are more tuned by the neighborhood neurons, a differential STDP is used to optimize local plasticity. Extensive experimental results on the benchmark MNIST (98.62%) and Fashion MNIST (89.05%) have shown that the proposed algorithm performs favorably against several state-of-the-art SNNs trained with backpropagation.

## 1. Introduction

Deep neural networks (DNNs) have been advancing the state-of-the-art performance in many domain-specific tasks, such as image classification (He et al., 2016), visual object tracking (Danelljan et al., 2015), visual object segmentation (Chen et al., 2017), etc. However, they are still far from the performance of efficiency and accuracy of information processing in the biological system. The structural connections (e.g., long-term feedback loops in the cortex) and functional plasticity (e.g., neighborhood plasticity based on discrete spikes) are carefully designed by the million years of evolution in the biological brain. This phenomenon has lead to the research of biologically plausible Spiking Neural Networks (SNNs). SNNs have received extensive research in recent years, and have a wide range of applications in various domains, such as brain function modeling (Durstewitz et al., 2000; Levina et al., 2007; Izhikevich and Edelman, 2008; Potjans and Diesmann, 2014; Zenke et al., 2015; Breakspear, 2017; Khalil et al., 2017a,b, 2018), image classification (Zhang et al., 2018a; Gu et al., 2019), decision making (Héricé et al., 2016; Zhao et al., 2018), object detection (Kim et al., 2019), and visual tracking (Luo et al., 2020). The discrete spike activation and high dimension information representation in SNNs make it more biologically plausible and energy-efficient. However, due to the non-differentiable characteristics, how to properly optimize the strength of synapses to improve the performance of the whole-brain network is still an open question.

Hebbian theory (Amit et al., 1994) could be considered as the first principle to demonstrate the relations between neurons, with the description of fire together, wire together. Later, Spiking Time Dependent Plasticity (STDP) (Bi and Poo, 1998) was proposed to model the synaptic plasticity. All the methods mentioned above are based on local adjustments without introducing global plasticity information.

**Figure 1:**
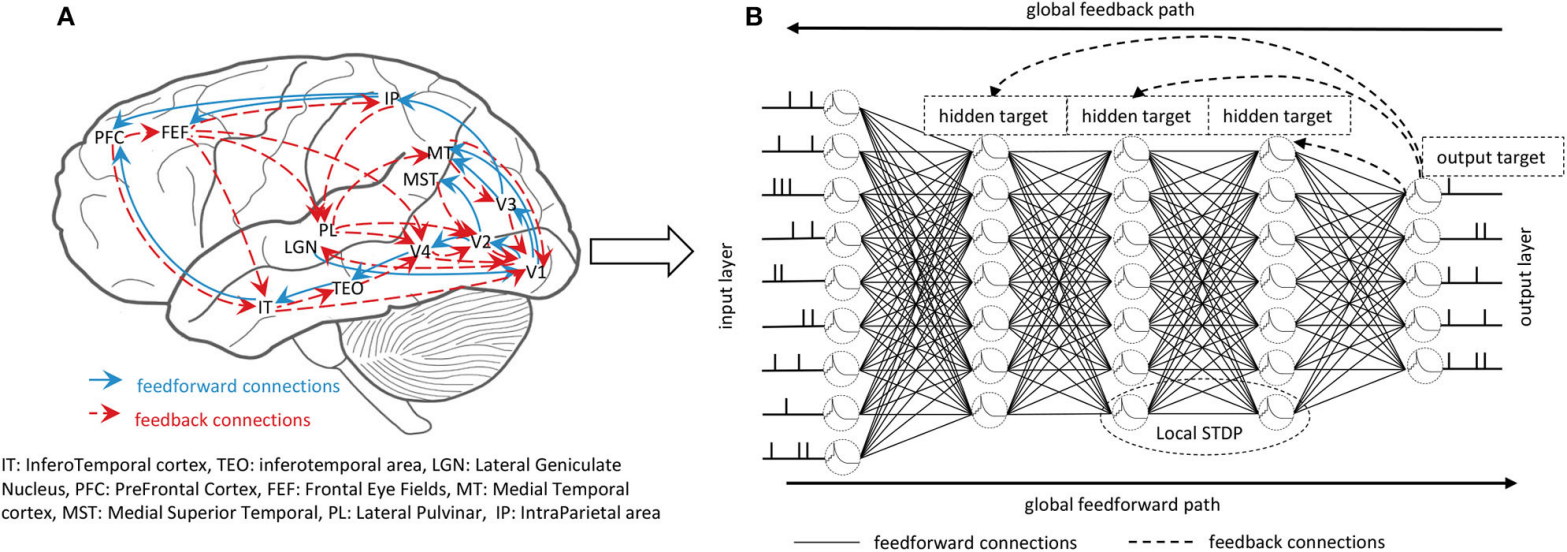
**(A)** The feedforward and feedback interactions in the brain. The massive feedback connections interact with feedforward connections contributing to the learning and inference of the brain. **(B)** The whole training process of the GLSNN. The global feedforward path uses the LIF spiking neuron model to get the forward state. The global feedback path uses the direct connection between the output layer and the hidden layers to propagate the target. The local STDP learning rule helps to update the weight of the neighborhood layers.

Learning and inference in the brain are based on the interactions of feedforward connections and mutual feedback connections across the hierarchy of cortical areas, as shown in Figure 1A. Both anatomical and physiological evidences point to the feedback connections in the brain (Felleman and Van, 1991; Sporns and Zwi, 2004). A large number of feedback connections in the cortex connect the feedforward series in the reverse order, thereby bringing global information from the higher cortex to the early cortical areas during perceptual inference. Feedback connections from higher layers will make predictions represented by the lower layers, and the feedforward path will get the state of neurons in the entire hierarchy. Therefore, combining global long-term feedback connections with local plasticity rules to train the SNNs is an urgent problem to be explored.

In this paper, we proposed an SNN training method that combines global feedback connections and local differential STDP learning rule and performs favorably against several existing state-of-the-art methods. The contributions of this paper are summarized as follows:

- We introduce the feedback connections in SNNs, which will help to introduce global plasticity information. The feedback connections are random, and no additional calculations are introduced.
- The global feedback connections combined with the local STDP plasticity rule are combined to directly optimize the synaptic strengths of all layers, instead of transferring error layer by layer as Back-Propagation. Compared with other methods, it provides an alternative method for training deeper SNNs.
- Extensive experimental results on different datasets indicated that the proposed algorithm could significantly improve the learning ability of SNNs.

## 2. Background

The success of DNNs is attributed mainly to the Back-Propagation algorithm (BP) (Rumelhart et al., 1986), which can take great advantage of the multilayer structure of neural networks to learn features related to a given task. However, firstly, the feedback path will have the symmetric weight of the forward path, which does not exist in biological systems, calling the weight transport problem (Lillicrap et al., 2016). Secondly, the precise derivatives of the operating point used in the corresponding feedforward path are needed. While for SNNs, information is transmitted in discrete spikes, and it is difficult to get the precise derivative of the operating point. Thirdly, the errors propagate layer by layer, which can easily lead to the problem of gradient vanishes or explosion. To tackle the problems mentioned above, many other learning rules are proposed to train the ANNs and further extended to train SNNs. In this section, we will review several of these approaches and several SNN frameworks in recent years.

### 2.1. Biologically Plausible Methods in ANNs

Recently, non-BP methods used to train neural networks can be roughly divided into three categories.

One family of promising approaches is Contrastive Hebbian Learning (Movellan, 1991). Equilibrium Propagation approaches (Scellier and Bengio, 2017) can be seen as a particular case of Contrastive Hebbian Learning. These kinds of energy-based models consist of two phases, the free phase is used to achieve the stationary distribution, and the clamp phase is used to update the network toward the target. Through the iteration of these two phases, the energy of the network can reach convergence gradually. However, due to the indirect feedforward process, the network state is obtained by minimizing the energy function. When the network becomes deeper, the entire algorithm will be unstable and therefore, difficult to train. We will give the experimental results below. Similarly, the free phase (feedforward propagation) and the clamp phase (feedback propagation) use the same weights, and the weight transpose problem still exists, as mentioned in backpropagation.

In order to solve the weight transport problem, the Random Feedback Alignment (RFA) algorithm (Lillicrap et al., 2016) uses a fixed random matrix *B* instead of the transposition of synaptic weights *W*, which can enable the network to converge to the optimal solution efficiently. Subsequent work DFA (Nøkland, 2016) propagates error signals through the direct connection matrix between the output layer and hidden layers. However, the error feedback does not influence the neural activity, which has not been confirmed by known biofeedback mechanisms based on neural communication.

In the Target Propagation (TP) family, for Difference Target Propagation (DTP) (Lee et al., 2015), targets for each hidden layer are passed through feedback connections, which avoids the weight transport problem, as the feedback connections are different from feedforward connections. The error-driven local representation alignment (LRA-E) (Ororbia and Mali, 2019), attempt to calculate the local target with the local error loss. Random feedback connections are utilized to transmit errors. However, the error is calculated and propagated layer by layer, and as the network deepens, performance will deteriorate.

### 2.2. Spiking Neural Networks

Much effort has been put into training SNNs, which can be roughly divided into three categories. First, directly convert the well-trained ANNs to SNNs. Second, SNNs are processed in some unique methods so that they can be trained with BP. Third, training SNNs with STDP and other biologically plausible methods.

For the conversion methods, SDBN (O'Connor et al., 2013) mapped an offline-trained deep belief network (DBN) onto an efficient event-driven SNN based on the Siegert approximation. The LIF response function is softened to lead to the bounded derivative value, which helps SDN (Hunsberger and Eliasmith, 2015) to convert the trained static network to a dynamic spiking network. WTSNN (Diehl et al., 2015) converted the DBNs into SNNs through weight and threshold balancing. Although these networks achieve good performance, the good results came from the well-trained ANNs, which does not reflect the characteristics of SNNs well.

For the BP training methods, DSN (O'Connor and Welling, 2016) proposed that SNN is equivalent to a deep network of ReLU units, and could be directly trained with BP. Event-SNN (Neftci et al., 2017) demonstrated an event-driven random BP rule for learning deep representations. SCSNN (Wu et al., 2019) used spike count as a surrogate for gradient backpropagation. BPSNN (Lee et al., 2016) treated the membrane potentials of spiking neurons as differentiable signals, which enabled the backpropagation. HM2-BP (Jin et al., 2018) proposed a hybrid macro/micro level backpropagation algorithm for training multi-layer SNNs. Temporal SNN (Mostafa, 2017) trained the SNN with temporal coding. STBP (Wu et al., 2018) trained the SNNs with BP both in spatial and temporal domains. The excellent performance of these methods came from BP, which turns out to not existed in the brain.

For STDP and other biologically plausible methods, Unsupervised-SNN (Diehl and Cook, 2015) trained an SNN with STDP, lateral inhibition, and an adaptive spiking threshold with a poor little performance 95% on the MNIST dataset. LIF-BA (Samadi et al., 2017) approximated dynamic input-output relations with piecewise-smooth functions based on fixed feedback weights. STCA (Gu et al., 2019) trained SNNs with credit assignments both in spatial and temporal domains. Both of them update the weights layer by layer. VPSNN (Zhang et al., 2018a) and Balance-SNN (Zhang et al., 2018b) trained the SNNs with Equilibrium Propagation, Balance-SNN is an improved version of VPSNN, which introduced much more learning rules to get the training balance of SNNs. However, as they trained with Equilibrium Propagation, the problems in Equilibrium Propagation also exist in both of them.

To sum up, a model to propagate the global plasticity information with a random feedback connection directly to each layer combined with the local plasticity learning rule to train SNNs has so far been rarely studied.

## 3. Methods

The pipeline of our model is shown in Figure 1B. First, we will introduce the spiking neuron model used in our framework. Second, the global and local plasticity learning process will be introduced. Third, the whole framework will be introduced to understand our model better.

### 3.1. The Basic LIF Neuron Model

**Figure 2:**
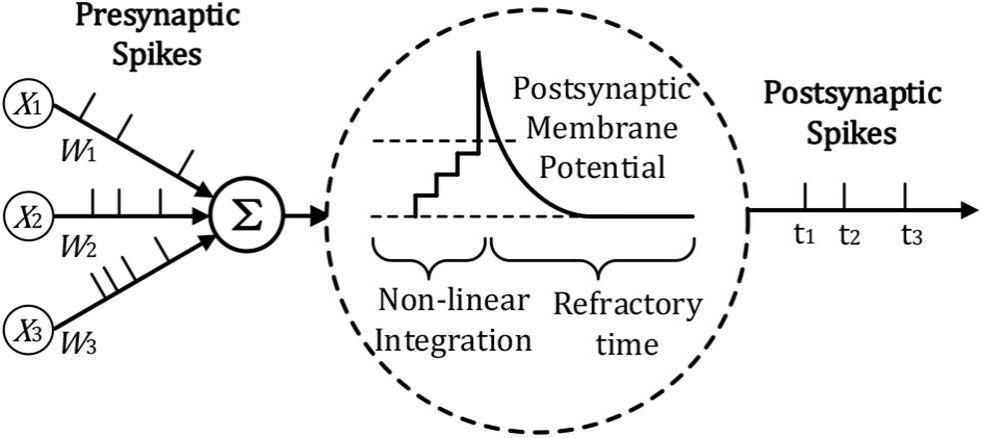
Illustration of LIF Neuron Model adopted from Lee et al. (2019) and Zhang et al. (2018a).

The spiking neuron model we use for temporal information processing is the Leaky integrate-and-fire (LIF) model, which is widely used in most SNN frameworks. As can be seen in Figure 2, for the LIF model, the neuron will accumulate the potential from the input, once its potential reaches the threshold, the neuron will be fired with a spike.

Generally, the membrane potential *V* can be calculated with Equation (1)

(1) $$\begin{array}{l} I\left(t\right)-\frac{V\left(t\right)}{R_{m}}=C_{m}\frac{dV\left(t\right)}{dt} \end{array}$$

*R*_*m*_ is the membrane resistance and *C*_*m*_ denotes the membrane capacitance. *I*(*t*) denotes the total input current from pre-synaptic neurons. For simplicity, we denote *V*(*t*) with *V*, *I*(*t*) with *I*, *g*_*L*_ and *V*_*L*_ denote leaky conductance and leaky potential. In a network with a more realistic synapse model, the input current *I* is generated as a change in conductance, which is caused by spikes of presynaptic neurons. The excitatory conductance *g*_*E*_ will be non-linearly increased by the number of the input spikes δ_*j*_ (Gerstner et al., 2014). *V*_*E*_ is the reversal potential from neuron *i* to neuron *j*. When the membrane reaches the threshold, the neuron will produce a spike, and the membrane will be reset to *V*_*reset*_. $\tau_{m}=\frac{C_{m}}{g_{L}}$, τ_*E*_ is the conductance decay of excitatory neurons, *w*_*j,i*_ is the synapse weight from neuron *j* to neuron *i*.

(2) $$\{\begin{array}{l} \tau_{m}\frac{dV_{i}}{dt}=-(V_{i}-V_{L})-\frac{gE}{gL}(V_{i}-V_{E})\\ \tau_{E}\frac{dg_{E}}{dt}=-gE+\sum_{j}^{N}w_{j,i}\delta_{j} \end{array}$$

### 3.2. The Global Plasticity Learning Process of Our Model

The global plasticity learning process is applied to a multi-layer feedforward neural network to illustrate better our learning algorithm, in which neurons in the previous layer are fully connected to the subsequent layer. In the adjacent layers, information from pre-synaptic neurons will be transferred to the post-synaptic neurons. For a deep spiking neural network, if only the spike is used, it will take a long time for the information transfer to the subsequent deeper layers, which will make the network hard to converge. To solve the problems, Diehl and Cook (2015) has used the spike trace to adjust the network weights, Zhang et al. (2018a) and Lee et al. (2016)'s work use voltage-based weight adjustments. Inspired by the residual neural network (He et al., 2016), which transfers the information as *x* + *f*(*x*), here we think that in addition to the spikes output by the LIF neuron can be used to regulate the weight, the input to the LIF neuron also contains a wealth of information. The final output of the neuron is denoted as *S*_*j*_(*t* + 1). To convert Equation (2) into discrete form, the whole process is shown in Equation (3):

(3) $$\{\begin{array}{l} V_{i}(t+1)=V_{i}(t)-\frac{dt}{\tau_{m}}[V_{i}(t)-V_{L}+\frac{gE}{gL}(V_{i}(t)-V_{E})]\\ g_{E}(t+1)=g_{E}(t)+\frac{dt}{\tau_{E}}(-g_{E}(t)+\sum_{j}^{N}w_{j,i}S_{j}(t+1))\\ \delta_{i}(t+1)=1\;V_{i}=V_{reset}\;if\;V_{i}>V_{th}\\ S_{i}(t+1)=\sum_{j}^{N}w_{j,i}S_{j}(t+1)+\tau \delta_{i}(t+1) \end{array}$$

τ is the constant to control the magnitude of the output. To accelerate the calculation, we only calculate the loss at the end of the simulation to update the target and weight. We denote the target with *S*^*T*^, *S*_*out*_ denotes the output of the last layer, *M* is the number of the samples. For the output layer, the loss function we choose here is the L2 norm so that the prediction error can be written as Equation (4):

(4) $$\{\begin{array}{l} loss=\sum_{i=1}^{M}∥S_{out}-S^{T}{∥}^{2}\\ e=2*\sum_{i=1}^{M}\left|S_{out}-S^{T}\right| \end{array}$$

Supposing a network with *L* layers. The output of the *l*_*th*_ layer is denoted with *S*_*l*_. For supervised learning, the target of the penultimate layer Ŝ_*L*−1_ can be directly calculated, as shown in Equation (5), *W*_*l*_ denotes the forward weight between the *l*_*th*_ layer and the (*l* + 1)_*th*_. η_*t*_ represents the learning rate of the target.

(5) $$\begin{array}{l} {Ŝ}_{L-1}=S_{L-1}-\eta_{t}\Delta S=S_{L-1}-\eta_{t}W_{L-1}^{T}e \end{array}$$

For the target of the other hidden layers, the target can not be directly calculated as Equation (5). By introducing the feedback connections, the prediction error can be easily transmitted to the hidden layers, and we denote the feedback layer as *G*_*l*_. Moreover, the target of the hidden layer can be written as Equation (6):

(6) $$\{\begin{array}{l} {\hat{S}}_{l}=S_{l}-G_{l}(e)\\ G_{l}(e)=B_{l}*e+b_{l} \end{array}$$

*B*_*l*_ denotes the random feedback weight of the *l*_*th*_ layer, and *b*_*l*_ represents the random feedback bias. With the operation of all layers, we can directly get the target of each layer.

### 3.3. The Local Learning Process of Our Model

STDP can be seen as the leading learning rule in the brain, and it can simulate the expected change of synaptic weights depending on states between pre-synaptic and post-synaptic (Bi and Poo, 1998), which can be regarded as a local learning rule. As introduced in (Xie and Seung, 2000; Hinton, 2007), STDP is associated with the change of postsynaptic activity. Here we use the difference between the feedforward state and feedback state to denote the change, as shown in Equation (7).

(7) $$\begin{array}{l} \Delta W\propto S_{j}S_{i}^{\prime }=S_{j}\left(S_{i}-{Ŝ}_{i}\right) \end{array}$$

where *S*_*j*_ and *S*_*i*_ indicate the pre-synaptic and post-synaptic output in the forward learning process. Ŝ_*i*_ denotes the target of the *i*_*th*_ layer calculated in Equation (6).

### 3.4. The Whole Learning Framework

For a multi-layer feedforward SNN, global plasticity information should be introduced so that STDP can train the whole network to obtain the desired result. Firstly, the feedforward process is used to obtain the feedforward state of the network, and then the feedback is used to obtain the targets of different hidden layers. Then, the change of weights in different neighborhood layers are calculated by local STDP plasticity rule in Equation (7). Finally, the weight of the forward propagation is updated with Equation (8):

(8) $$\begin{array}{l} W=W-\eta_{w}\Delta W \end{array}$$

η_*w*_ denotes the learning rate of weight.

**Figure 3:**
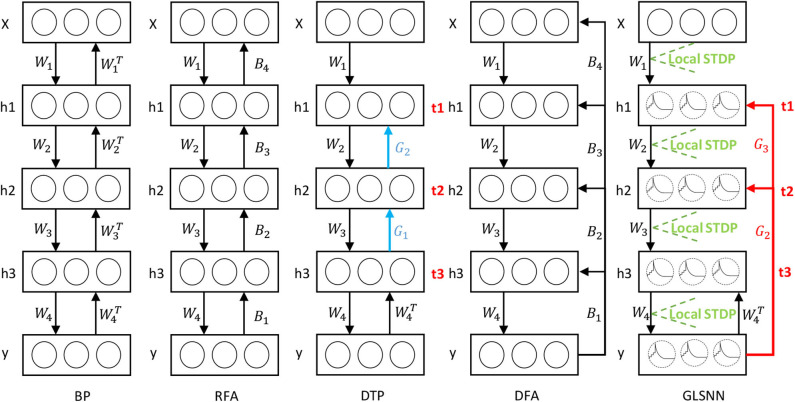
The learning process of our GLSNN compared with BP, RFA, DTP, and DFA. B in RFA and DFA means the random matrix to transfer the error directly. Blue connection *G*_*l*_ in DTP means the feedback layer needs to update. Red connection *G*_*L*_ in GLSNN means the feedback layer without updates.

Inspired by FAs (Lillicrap et al., 2016; Nøkland, 2016), random weights can be used to transmit the error in the network. In this paper, we use the random feedback layer to get the target of the hidden layers. As shown in Figure 3, in our model, feedback connections are directly connected from the output layer to the hidden layers, which means that the neural network can update the parameters of all hidden layers simultaneously, and the random feedback connections do not introduce extra computations. The details are shown in Algorithm 1.

**Algorithm 1:** The whole learning process of our GLSNN.

**Require:** Initialize a multi-layer neural network with *L* layers	
	Feedforward process in Equation (3) *F*_*i*_
	Feedback process in Equation (6) *G*_*i*_
	t = 0, simulation interval *dt*, and simulation time T, max iteration *EPO*
1:	**for** epoch = 1 to *EPO* **do**
2:	**while** *t* ≤ *T* **do**
3:	**for** i = 1 to L-1 **do**
4:	*S*_*i*_=*F*_*i*_(*S*_*i*−1_).
5:	t = t + dt
6:	**end for**
7:	**end while**
8:	Get the prediction error *e* with Equation (4).
9:	Get the target of the penultimate layer Ŝ_*L*−1_ with Equation (5).
10:	**for** i = 1 to L-2 **do**
11:	Ŝ_*i*_=*S*_*i*_−*G*_*i*_(*e*)
12:	**end for**
13:	**for** i = 1 to L-1 **do**
14:	Update synapse weights Equations (7) and (8)
15:	**end for**
16:	**end for**

## 4. Experiments

In this section, we experimentally evaluate the performance of our model on two benchmark datasets, basic MNIST (LeCun, 1998) and Fashion MNIST (Xiao et al., 2017). The experiments are performed with PyTorch on TITAN RTX. To fully reflect the performance of our algorithm, the fully connected network is considered to carry out the experiment without batch normalization or weight regularization. The update method of the weight is the Stochastic Gradient Descent (SGD) method. In addition, we compare our GLSNN with other state-of-the-art biological plausible methods. The initiation method of the weight is the same as DTP (Lee et al., 2015). Also, the ablation studies are performed to study the effect of the feedback layers. For the parameters of the network, the learning rate for the target η_*t*_ = 0.5, the learning rate for the weight η_*w*_ = 0.015. The batchsize is 10. For the hyper-parameter of the LIF neuron as described in section 3, we set *V*_*E*_ = 0.2, *V*_*I*_ = 0, *V*_*L*_ = 0, *V*_*th*_ = 0.0009, *V*_*reset*_ = 0, τ_*m*_ = 0.5, τ_*E*_ = 0.2, τ = 0.01, *g*_*leak*_ = 20, the simulated time interval *dt* = 0.01, and the total simulation time *T* = 0.1.

### 4.1. MNIST

MNIST is the most widely used dataset to measure the performance of the algorithm in machine learning. It consists of 60,000 training samples and 10,000 test samples, used to describe the hand-written digits from 0 to 9. The sample size is 28*28. The number of epochs is set with 100. We wonder how our model fares in this benchmark as the model goes deeper in that target is directed computed from the output layer. To that end, we have trained a network of 3 hidden layers of different hidden neurons to evaluate the performance of the network.

**Figure 4:**
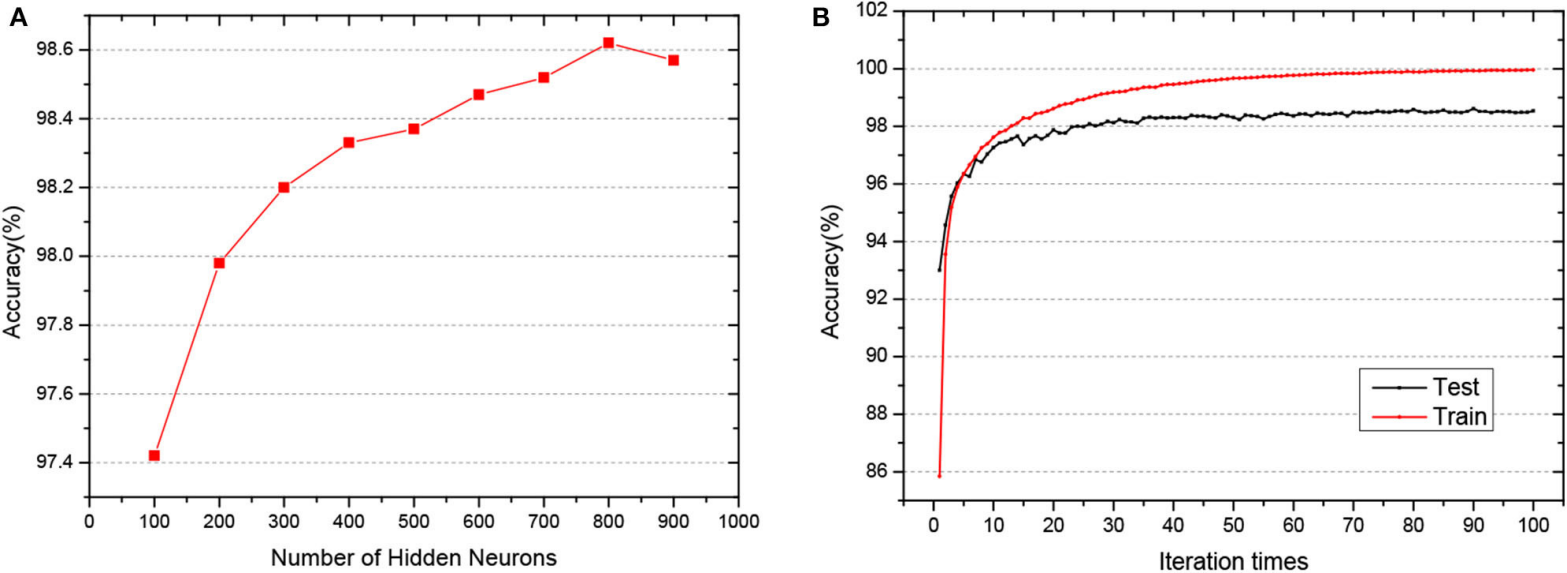
**(A)** The test accuracy of GLSNN of different hidden neurons of 3 hidden layers. **(B)** The train and test accuracy when the hidden layer is set with 800*3.

**Figure 5:**
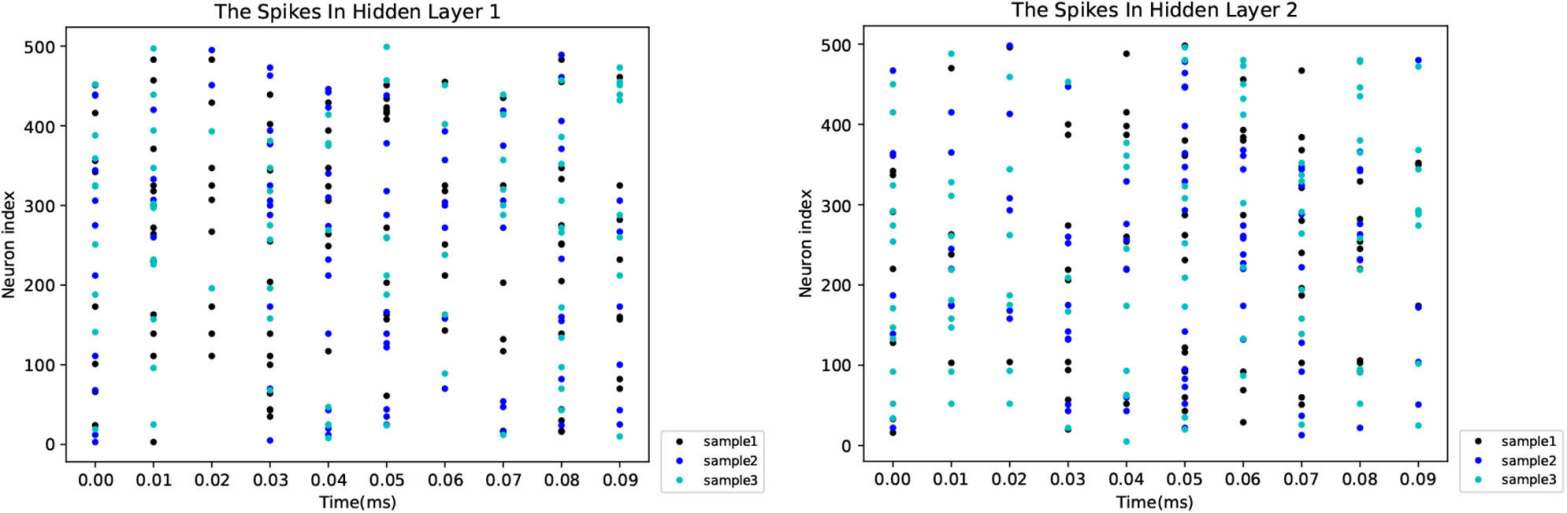
The spikes in the hidden layer of the three randomly chosen samples.

As shown in Figure 4, when the network structure is set with [784-800-800-800-10], the test accuracy is the highest at 98.62%. To demonstrate the superiority of our GLSNN, we compare our methods with several different SNN frameworks, as can be seen in Table 1, our GLSNN has surpassed all other SNN frameworks trained without BP, such as Unsupervised-SNN (Diehl and Cook, 2015), VPSNN (Zhang et al., 2018a), and so on. Moreover, for the BP trained SNNs, we have exceeded most of them. For the Balance-SNN (Zhang et al., 2018b), in addition to the STDP learning rule, several other rules were introduced, such as LTP, LTD, STF, STD, however only 0.2% accuracy improved compared to our GLSNN. For SCSNN (Wu et al., 2019), BPSNN (Lee et al., 2016), HM2-BP (Jin et al., 2018), and STBP (Wu et al., 2018), the different levels of backpropagation was connected to contribute to their superior performance, however, which is non-existent in the human brains. To the best of our knowledge, our result could be a new record for the SNNs trained with STDP. The spike transfer process is shown in Figure 5, as the network structure is set with [784-500-500-10].

**Table 1:** Comparison of classification accuracies of GLSNN with other SNN frameworks on the MNIST dataset.

**Model**	**Structure**	**Neural coding**	**Learning rule**	**Acc**
SDBN (O'Connor et al., 2013)	FC	Spike	ANN to SNN	94.09
Unsupervised-SNN (Diehl and Cook, 2015)	FC	Spike	STDP	95
LIF-BA (Samadi et al., 2017)	FC	Spike	Broadcast Alignment	97.05
SN (O'Connor and Welling, 2016)	FC	Rate	BP	97.93
Event-SNN (Neftci et al., 2017)	FC	Rate	BP	97.98
Temporal SNN (Mostafa, 2017)	FC	Spike	BP with Temporal Coding	98
SDN (Hunsberger and Eliasmith, 2015)	FC	Spike	ANN to SNN	98.37
VPSNN (Zhang et al., 2018a)	FC	Spike	Equi-prop + STDP	98.52
STCA (Gu et al., 2019)	FC	Spike	Spatial + Tempral Credit Assignment	98.6
**GLSNN (This study)**	**FC**	**Spike**	**Global Feedback + STDP**	**98.62**
Balance-SNN (Zhang et al., 2018b)	FC	Spike	Equi-Prop + Multiple Balance Rules	98.64
SCSNN (Wu et al., 2019)	FC	Rate	BP	98.66
BPSNN (Lee et al., 2016)	FC	Rate	BP	98.71
HM2-BP (Jin et al., 2018)	FC	Rate	Macro/Micro level BP	98.88
STBP (Wu et al., 2018)	FC	Rate	Spatial + Tempral BP	98.89

**Figure 6:**
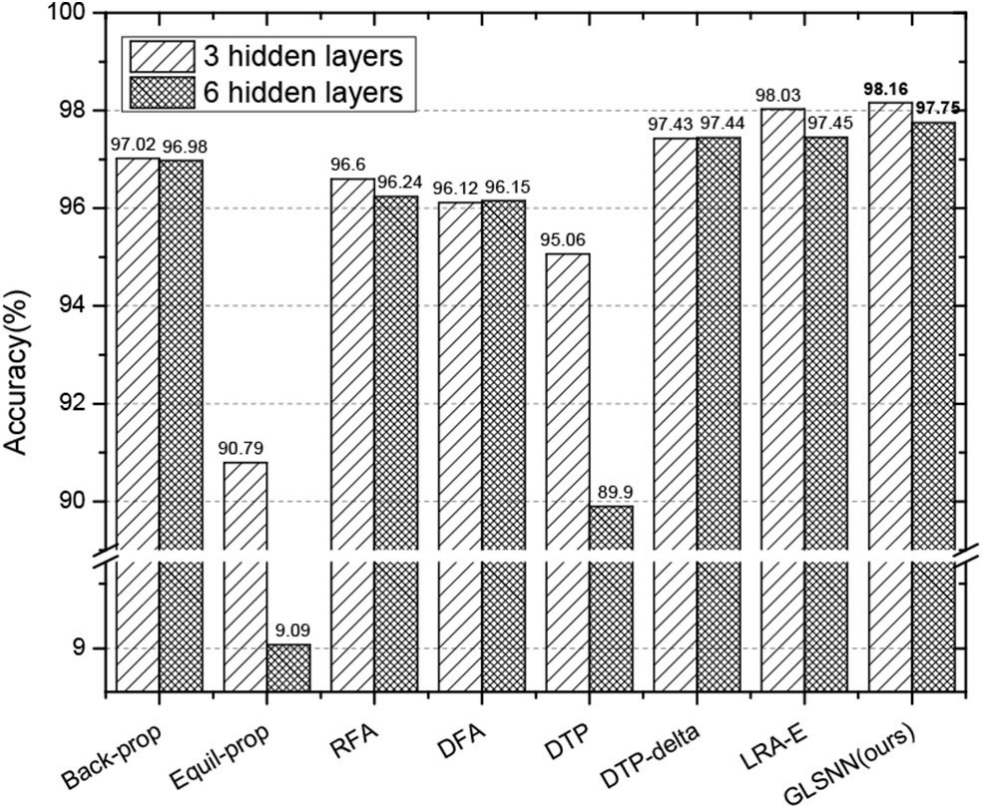
The test accuracy on MNIST dataset of GLSNN compared with ANNs trained with BP, Equil-Prop, RFA, DFA, DTP, DTP-delta, and LRA-E with different hidden layers.

Also, to prove that our algorithm still performs well when the network is going deeper, we test the results with different hidden layers, whose hidden neurons are set with 256 for consistency with the paper (Ororbia and Mali, 2019). As can be seen in Figure 6, for Equil-prop methods, the accuracy quickly drops down when the network is deeper. Also, the accuracy of the DTP method begins to struggle from 95.06 to 89.9%, which shows the instability of them. Compared with other stable methods, our GLSNN outperforms better than them both for the five hidden layers and the eight hidden layers, which indicates the stability and superiority of our algorithm.

Also, to measure the computation speed of our model, we test the average runtime per epoch with different hidden layers as shown in Table 2.

**Table 2:** The average training time (seconds) per epoch.

500 1	500 2	500 3	500 4	500 5	500 6
80.86	124.99	133.33	178.25	200.08	256.98

**Figure 7:**
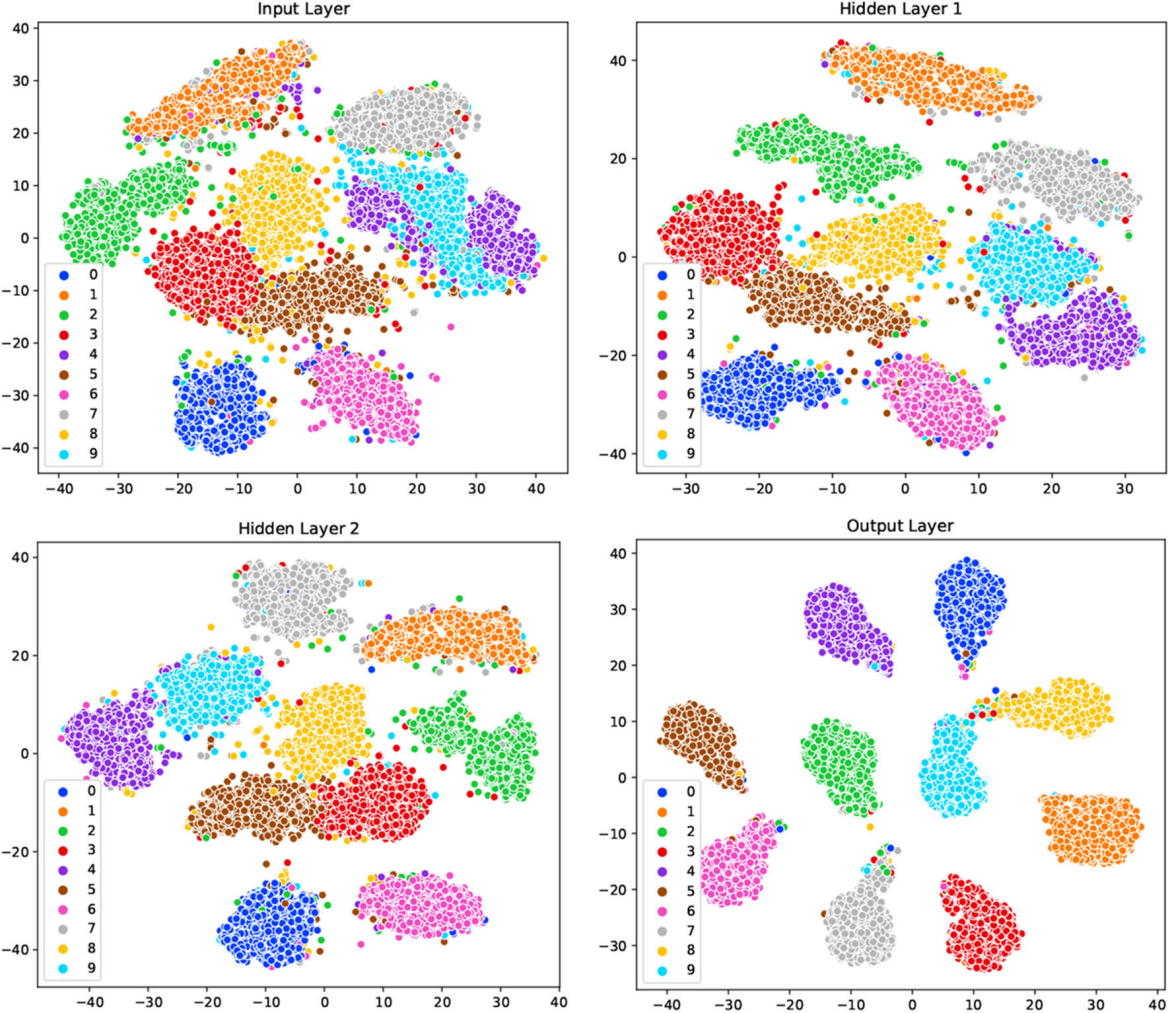
The visualization on the input layer, hidden layer 1, hidden layer 2, and output layer in GLSNN with t-SNE.

To demonstrate the underlying mechanism of our GLSNN model, the t-SNE method (Maaten and Hinton, 2008) was used to visualize the model's clustering ability of different layers. The network structure is set with [784-500-500-10], as shown in Figure 7, for the original input, samples of different categories are very close to each other, and some clusters contain samples from other categories. After the training of SNN, the separability of the output information of the hidden layer shows more vital clustering ability than the input layer as the interval between the class clusters is coming larger. For the output layer, different categories are distinguished, which has shown that the learning process of our GLSNN has helped the network to perform better clustering and classification performances.

### 4.2. Fashion MNIST

**Figure 8:**
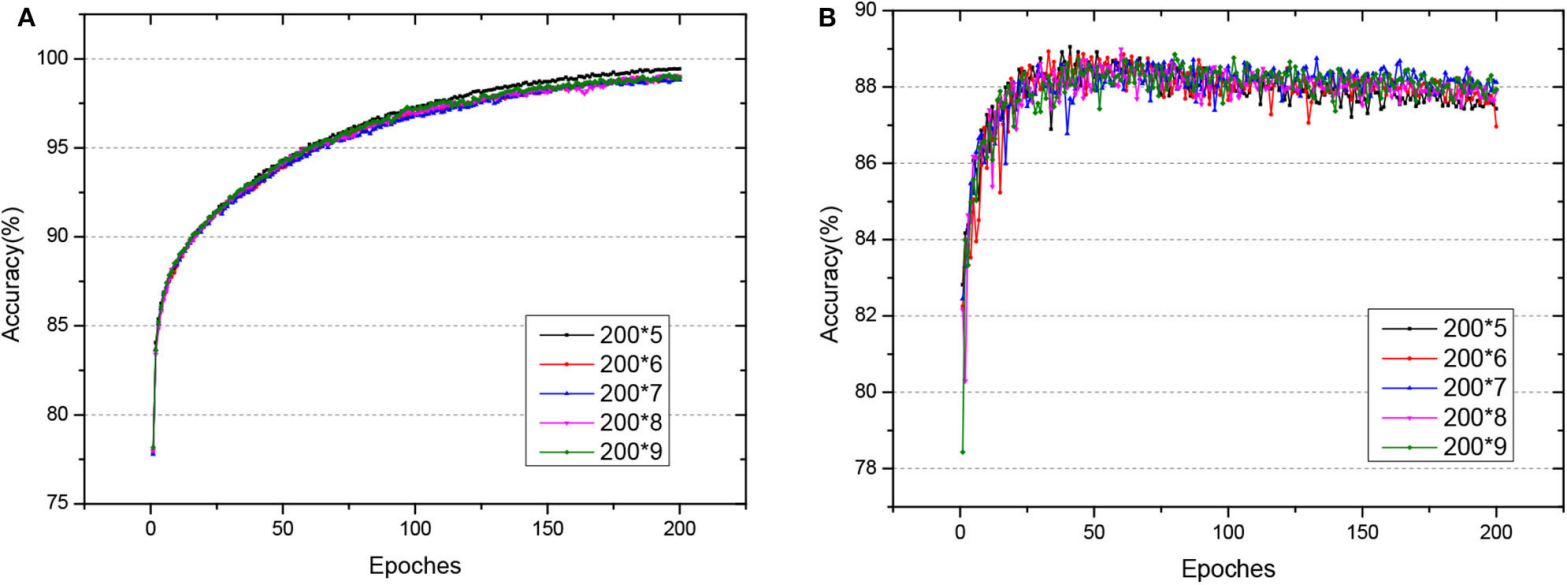
**(A,B)** The train and test accuracy of GLSNN of different hidden layers of Fashion MNIST, the 200*n, means n hidden layers with 200 neurons each hidden layer.

Fashion-MNIST is a more complex version compared to MNIST, consisting of gray-scale images of clothing items. Since the dataset is more complicated compared with MNSIT, the training epoch is set with 200, and we tried networks of different hidden layers, as shown in Figure 8. When the network structure is set with five hidden layers of 200 hidden neurons each layer, the network achieves the best performance with 89.05% accuracy on the test dataset. Also, we compare our GLSNN with other biologically plausible methods shown in Table 3. We have chosen the best results of each method as recorded in (Ororbia and Mali, 2019). Our GLSNN exceeds all of them.

**Table 3:** The test accuracy on the Fashion MNSIT dataset of GLSNN compared with VPSNN and other ANNs trained with BackProp, Equi-Prop, RFA, DFA, DTP, DTP-delta, and LRA-E.

**Model**	**Structure**	**Type**	**Performance**
VPSNN (Zhang et al., 2018a)	FC	SNN	82.69
Equiprop (Scellier and Bengio, 2017)	FC	ANN	85.99
DTP (Lee et al., 2015)	FC	ANN	86.4
DTP_delta (Ororbia and Mali, 2019)	FC	ANN	87.01
LRA-E (Ororbia and Mali, 2019)	FC	ANN	87.69
RFA (Lillicrap et al., 2016)	FC	ANN	88.01
DFA (Nøkland, 2016)	FC	ANN	88.41
Backprop (Rumelhart et al., 1986)	FC	ANN	88.45
**GLSNN (This study)**	**FC**	**SNN**	**89.05**

### 4.3. Ablation Studies

To study the effect of the feedback layers of the network, we create four networks with 7, 8, 10, and 12 layers separately. All of the hidden neurons are set with 200. First, we remove all the feedback connections of the network, which means only the weight of the last two-layers could be updated. Then we incrementally add the feedback layers in the network to see the performance of the network.

**Figure 9:**
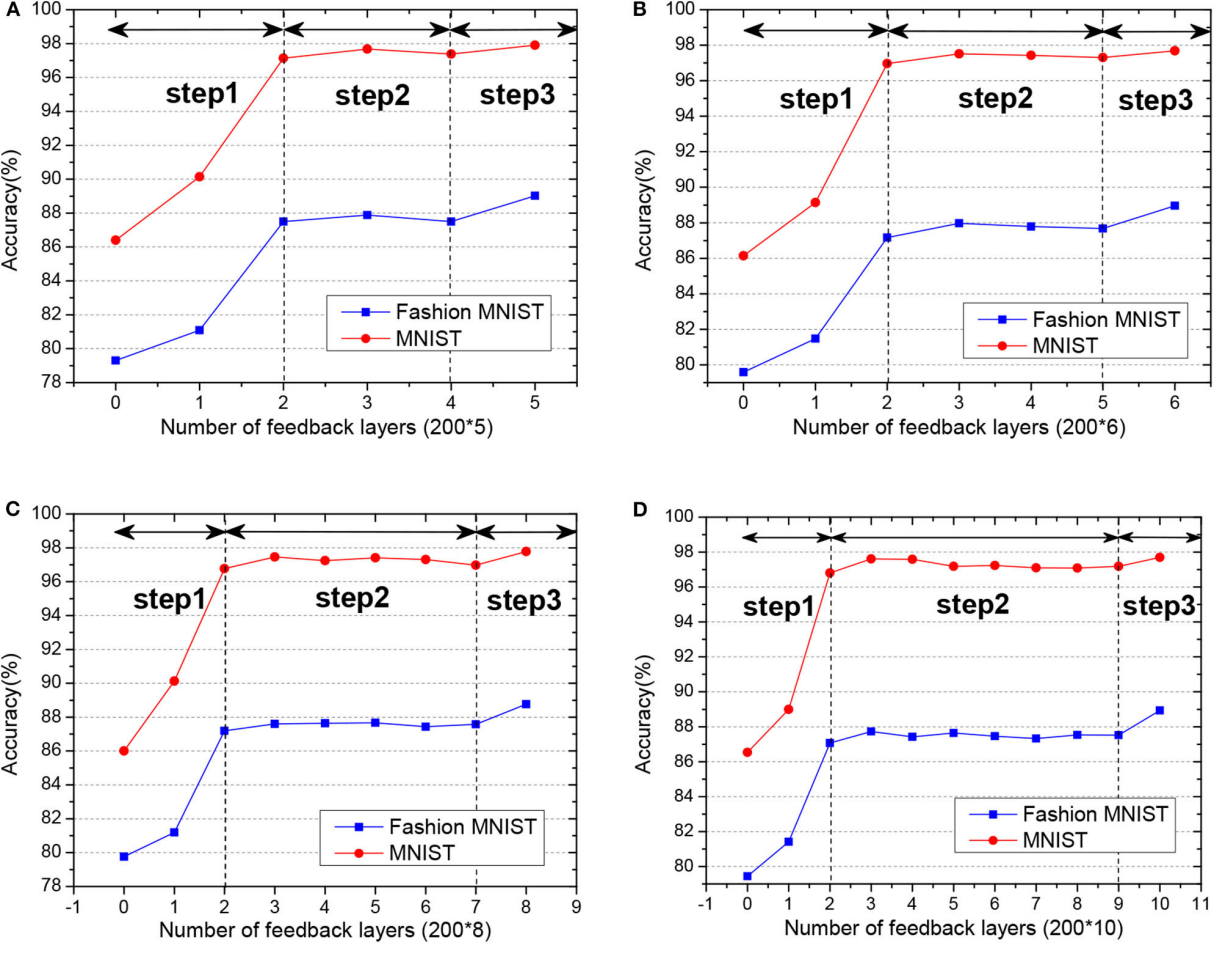
**(A–D)** The test accuracy of GLSNN with different feedback layers on MNIST and Fashion MNIST, and the variation in accuracy can be roughly divided into three steps.

As shown in Figure 9, with the increase of the number of feedback layers, the performance of the network gradually improves. When all the feedback layers are added, the SNN reaches the highest accuracy. The performance of the network did not improve linearly with the increase of the feedback layers. The variation in accuracy can be roughly divided into three steps:

- In step 1, the linear increment of accuracy with weights tuning in only top layers.
- In step 2, the non-increment or stabilization of accuracy with weight tuning in both top and mid-layers.
- In step 3, the prominent increment toward the best accuracy with only adding into the weight tuning in the bottom layer.

The deeper layers play a role in decision-making, while the former layers play a role in feature extraction. That is to say, the feedback connections play a significant role in the perceptual inference, which is consistent with neurophysiology (Harris and Shepherd, 2015).

### 4.4. Comparison With Other Traditional SNNs Trained With STDP

For the SNNs trained with STDP, the problem is how to introduce global information. The success of the BP algorithm in deep neural networks training is mainly due to the chain rules, which introduce the global error. Traditional SNNs trained with STDP often sidestep this problem, that is they avoid multi-layer training. For Diehl's unsupervised SNN (Diehl and Cook, 2015), only the weight between the input and excitatory neurons is trained with STDP. The extension (Hao et al., 2020) modified the last clustering layer to a supervised classification layer. Masquelier (Masquelier and Thorpe, 2007) introduced a multi-layer SNN combined with convolutional/pooling layer, feature discovery layer and a classification layer. However, the first convolutional layer is set with the Gabor filters, and only the feature discovery layer is trained with STDP. To solve this, Tavanaei (Tavanaei and Maida, 2017) introduced a sparse coding model to replace the handcrafted features in Masquelier and Thorpe (2007). However, the training is layer-wise, the feature discovery layer can only be trained after the first convolutional layer is completed training. Recently, Zhang's work (Zhang et al., 2018a) introduced the equilibrium propagation, the forward and feedback process in SNNs are implicitly defined in the negative and positive phase in equilibrium propagation, which solved the multi-layer training in SNNs to a certain extent. However, due to the implicit definition, when the network went deeper, it becomes hard to converge to a stable situation. Our GLSNN explicitly introduced the global feedback connections, which provides a feasible solution to the training of the multi-layer SNN.

## 5. Conclusion and Future Work

In this paper, we propose an SNN training method, which takes full advantage of the global and local plasticity information. We mimic the global feedback connections and the local STDP learning rules in the brain, providing a powerful way to train a multi-layer SNN. The global random feedback connections help to propagate the target from the output layer to the hidden layers. The local STDP learning rule is utilized to optimize the local synaptic strength of the network with the obtained target. Our GLSNN offers an alternative way to solve the weight transpose problem in BP, as well as the feedback layers are directly connected to the hidden layers, leading the weight of each layer can be directly updated without the error transmitted layer by layer. Experiments indicate that our GLSNN model has performed favorably against several state-of-the-art SNNs on the standard benchmark MNIST and Fashion MNIST dataset.

In terms of future work, the authors intend to study more biologically inspired learning rules in this work, as we only use the STDP local learning rule. The dynamic combination of different learning rules and different types of spiking neurons may further enhance the learning performance of the network. Also, we only verify the performance on the fully connected network structures, in the following work, we would consider more complex network structures such as convolutional neural network and recurrent neural network to accommodate more complex visual perception tasks, such as video object detection and visual tracking.

## Data Availability Statement

Publicly available datasets were analyzed in this study. This data can be found here: http://yann.lecun.com/exdb/mnist/; https://github.com/zalandoresearch/fashion-mnist.

## Author Contributions

DZ and YZ designed the study, performed the experiments and the analyses. MS and FZ participated in the biological background discussion and refined the paper. DZ, YZ, and TZ were involved in algorithm discussion, result analysis, and wrote the paper. All authors contributed to the article and approved the submitted version.

## Conflict of Interest

The authors declare that the research was conducted in the absence of any commercial or financial relationships that could be construed as a potential conflict of interest.
